# Antidepressant and neuromodulatory potential of hydroalcoholic extract of *Helianthus annuus* florets in mouse models of depression

**DOI:** 10.1515/tnsci-2025-0376

**Published:** 2025-06-24

**Authors:** Km Kajal, Tarique Anwer, Ankit Verma, Mohammad Firoz Alam, Saeed Alshahrani, Muhanad Alhujaily, Mohammed Naffaa Alruwaili, Ali Assiry, Abdullah Saleh Salem Alrashah

**Affiliations:** Department of Pharmacy, HIMT College of Pharmacy, Dr. A.P.J Abdul Kalam Technical University (AKTU), Knowledge Park 1, Greater Noida, Gautam Budh Nagar, U.P., 201310, India; Department of Public Health, College of Applied Medical Sciences, University of Bisha, P.O. Box 225, Bisha, 67714, Saudi Arabia; Department of Pharmacology & Toxicology, College of Pharmacy, Jazan University, 114, Jazan, Saudi Arabia; Department of Medical Laboratory Sciences, College of Applied Medical Sciences, University of Bisha, P.O. Box 225, Bisha, 67714, Saudi Arabia; Forensic Toxicology Services Unit, Northern Borders Region, Ministry of Health, Arar, 73241, Saudi Arabia; Mohayil Hospital, Health Affairs of Aseer, Abha, 63711, Saudi Arabia; King Khalid Hospital, Najran, 66251, Saudi Arabia

**Keywords:** *Helianthus annuus*, depression, oxidative stress, inflammatory markers, brain monoamines

## Abstract

**Background:**

Depression is a pervasive neuropsychiatric disorder having significant social and economic impacts and often linked to imbalances in neurotransmitter systems. Traditional herbal medicines have garnered attention for their potential antidepressant effects, with limited research on *Helianthus annuus* (sunflower) as a therapeutic option.

**Objectives:**

The present study was carried out to investigate the anti-depressant and neuromodulatory potential of hydroalcoholic extract of *Helianthus annuus* (*H. annuus*) florets in mouse models of depression.

**Methods:**

Depression was induced in rats by the forced swim test (FST) and tail suspension test (TST). The hydroalcoholic extract of *H. annuus* was used as the test drug given in the doses of 200 and 400 mg/kg, whereas fluoxetine was used as the standard drug.

**Results:**

The results revealed that the *H. annuus* extract decreased the immobility time significantly as reflected in FST and TST. Treatment with *H. annuus* extract also demonstrated significant improvement in the swimming and climbing times as reflected in FST. Administration of *H. annuus* extract significantly improved neurotransmitter levels such as serotonin, dopamine, and norepinephrine, which were significantly lowered in depression control rats. The mean value of thiobarbituric acid reactive substances was significantly lowered after the administration of *H. annuus* extract. Additionally, the levels of glutathione, superoxide dismutase, and catalase were significantly increased after the administration of *H. annuus* extract. Additionally, the mean value of inflammatory cytokines, for example, tumour necrosis factor alpha and interleukin-6 were reduced significantly in groups treated with *H. annuus* extract.

**Conclusions:**

The results suggest that *H. annuus* extract exhibited significant antidepressant and neuromodulatory potential by ameliorating behavioural parameters, oxidative stress, and inflammatory markers.

## Graphical abstract

**Figure:**
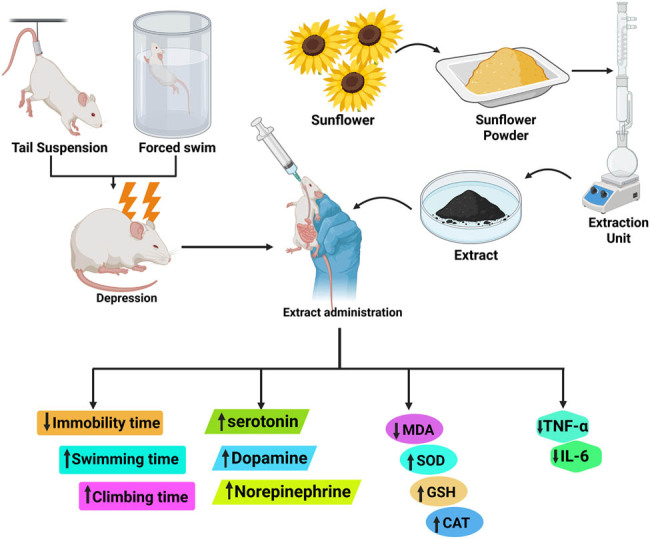

## 1 Introduction

Major depressive disorder (MDD) is a diverse collection of neuropsychiatric disorders linked to high rates of morbidity, death, and functional impairment. The Diagnostic and Statistical Manual of Mental Illnesses has recently identified depression mood, loss of interest or pleasure, and other symptoms such as increased appetite or weight gain, sleeplessness or excessive sleep, tiredness, excessive or inappropriate guilt, a sense of worthlessness, difficulty in concentrating, psychomotor hyperactivity or inertia, suicidal thoughts, and indecision as hallmarks of MDD [1]. Alterations to the physiologic, behavioural, emotional, motivational, psychomotor, and cognitive processes are also the reflected symptoms [2]. It also has an impact on the community’s standard of living and a significant contributor factor to high suicidal rates. In general, the symptoms must cause clinically significant distress or impairment in social, occupational, or other areas of functioning [3]. There are numerous subtypes of this complicated mood illness, as well as diverse aetiology, and symptoms that can vary from mild to severe or no psychotic symptoms [4]. Major depressive episode prevalence rates are high and varied: in Japan, they range from 3 to 17%; in the United States, they fall between 8 and 12%; in North America, they vary from 5 to 8%, whereas in India the mean rate was estimated to be 65 per 1,000 individuals [5]. Women are more vulnerable than men, according to several studies. There is no single cause for depression, rather it is a combination of genetic, physiological, environmental, and psychological factors. The fact is that some but not all forms of depression run in families, which highlights the connection between gene–gene and gene–environment [6]. By 2030, it will be considered as the second most common cause of death. Many theories have been proposed over the years regarding the pathological causes of depressive disorders, including interrupted circadian rhythms, neurotransmitter imbalances, malfunctioning of GABA (gamma aminobutyric acid), glutamate signalling, and modified HPA (hypothalamic–pituitary–adrenal) axis activity. The latter, also known as the “Monoamine Hypothesis,” has been the subject of most research studies and has been the most widely accepted hypothesis since 1960.

The most effective treatment strategy is still the pharmacological modification of monoamine transmission, although this theory alone is insufficient to explain depression [7]. Treatment with clinically available antidepressants, such as monoamine oxidase inhibitors like selegiline, selective serotonin reuptake inhibitors like fluoxetine, norepinephrine (NE) reuptake inhibitors like amitriptyline, atypical antidepressants like trazodone, and others, is therefore frequently coupled with increased side effects or undesirable consequences such as feeling nauseated, throwing up, digestive pain, problems with sexuality, and decreased appetite [8]. However, these drugs are effective for only a subset of patients. Excitement, anxiety, restlessness, seizures, impaired vision, orthostatic hypotension, hypertensive crisis, insomnia, and restlessness are occasionally experienced by some patients. These symptoms collectively impair cognition and behaviour [9]. Therefore, scientists are looking for an alternative antidepressant from natural sources that has been used traditionally and whose safety profile is well established, in order to achieve better therapeutic benefits with fewer and mild adverse reactions. The purpose of the present study is utilizing an animal model of depression to investigate various traditional medicinal plants or identify active phytoconstituents for potential antidepressant properties [10,11,12,13].

*Helianthus annuus*, renowned for its vibrant blooms and prized sunflower seeds, has disk florets that after pollination develop into seeds. Interestingly, these disk florets are prominent features of sunflower’s reproductive process [14]. Although sunflower seeds are a promising protein source for human diet, they are primarily used for the extraction of oil, since sunflower seed oil has attractive value in the food industry. It is rich in vitamin E and hence offers high nutritional benefits [15]. Numerous chemicals found in *H. annuus*, including flavonoids, sterols, saponins, and unsaturated terpenoids, have been identified through phytochemical investigations [16]. Previous research indicates that flavonoids and their analogues selectively interact with central benzodiazepine receptors, causing animal models to exhibit effects similar to those of benzodiazepines. Furthermore, it has been demonstrated that compounds such as terpenoids and saponins alter the amounts of neurotransmitters like dopamine (DA), noradrenaline (NA), and serotonin (5-HT) [13,17,18].

This research is focused on addressing the existing knowledge gap of the effects of *H. annuus* flower extract and its constituents on the central nervous system. This research study is intended to investigate the antidepressant and neuromodulatory potential of hydroalcoholic extract of *H. annuus* florets in mouse models of depression, providing insight into their possible medicinal uses.

## 2 Materials and methods

### 2.1 Animals used in experiment

Wistar male rats of weight 150–250 g were purchased from AIIMS, New Delhi. The rats were acclimatized for 7 days under controlled environmental conditions and maintained at 25°C with a 12-h light/dark cycle. During this period, they had free access to standard food and water.

### 2.2 Collection and authentication of sunflower (ray florets and disk florets)

*H. annuus* (ray florets and disk florets) were collected from the botanical garden of Noida, during the month of January 2024. Following collection, the authentication of sample was done at the BGIR, Pusa Road, New Delhi (authentication no. BSI/BGIR/1/TECH/2023/65). The collected flowers (ray florets and disk florets) were kept in a shed for 10 days for the purpose of drying.

### 2.3 Preparation of the extract

A 2.5 L aspirator jar was properly cleaned, dried, and filled with 500 g of dried floret powder. The aspirator was then filled with hydroalcoholic solvent – ethanol (90%) and water in a ratio of 70:30. The Soxhlet apparatus was properly connected, and the extraction process was carried out at 60°C for 12 h for complete extraction. Afterwards, the evaporation process was carried out using a rotary evaporator to remove the solvent content from the extract. The remaining extract after solvent evaporation was stored in an air tight container at refrigerator temperature (2–8°C).

### 2.4 Preliminary phytochemical analysis

**Figure 1:**
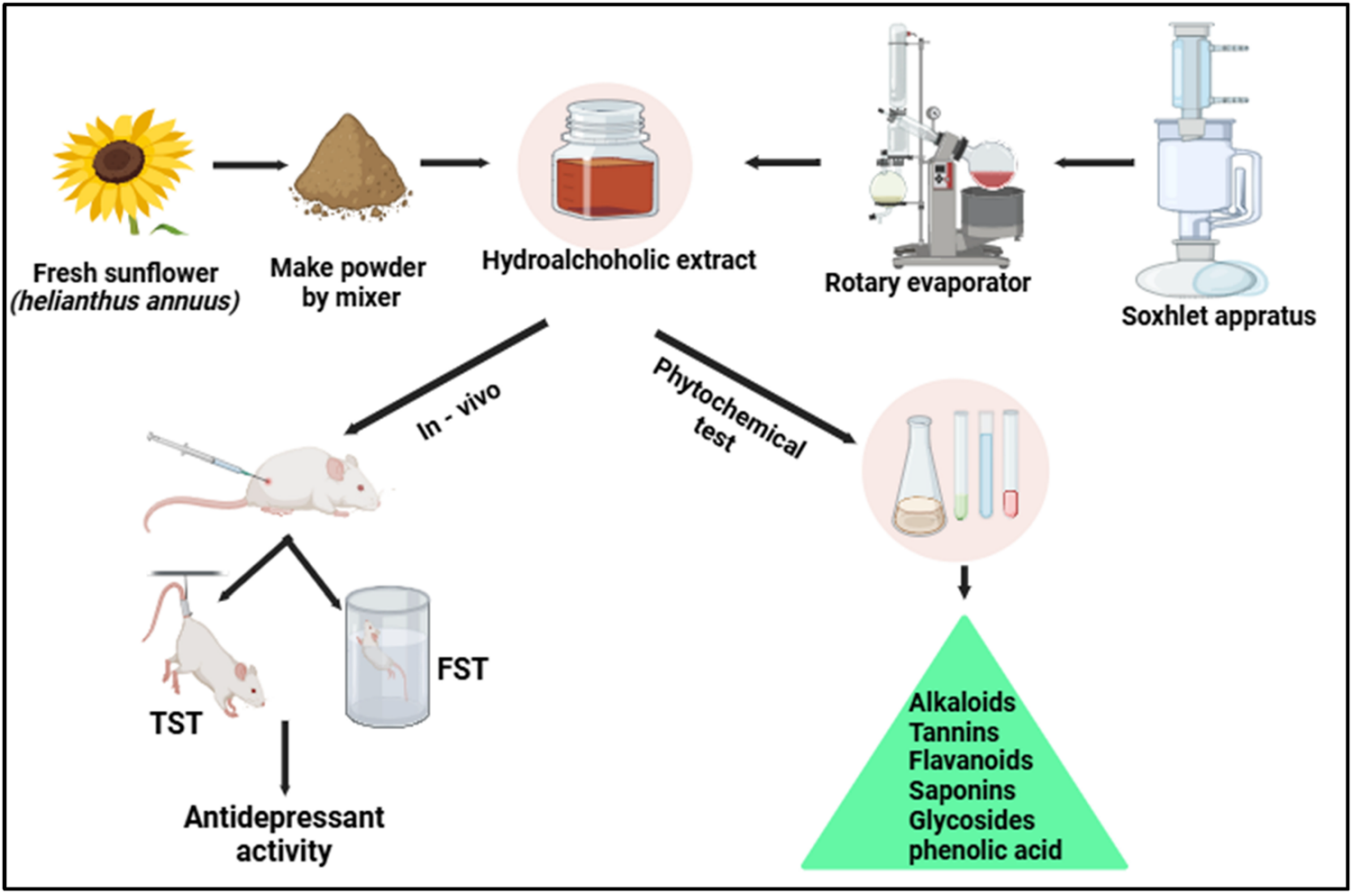
Plant extraction and phytochemical analysis.

Qualitative phytochemical assays were performed on the ethanolic extract of *H. annuus* to identify various compounds, including triterpenoids, alkaloids, carbohydrates, and flavonoids (Figure 1).

### 2.5 Drug and chemicals

Fluoxetine powder was procured from Hari Ganesh Pharma Private Ltd, Hyderabad, India. All other chemicals like BSA, EDTA, DTNB, trichloroacetic acid, thiobarbituric acid, Folin phenol reagent, nitroblue tetrazolium (NBT), oxidized glutathione (GSH), and reduced GSH were procured from Sisco Research Laboratories Pvt. Ltd. Cytokine kits for tumour necrosis factor alpha (TNF-α) and interleukin-6 (IL-6) were purchased from Krishgen Biosystems, Mumbai, India.

### 2.6 Treatment schedule and experimental design

Rats were assigned into a total of five groups, each consisting of six rats. Drugs were administered orally according to the weight of the animal. Group-1, considered as normal control in which normal saline was administered through the oral route for 15 days. Group-2, considered as depression control in which only normal saline was administered for 15 days followed by forced swim test (FST) and tail suspension test (TST) to induce depression. Group-3, considered as test drug treatment-1, in which *H. annuus* floret extract was administered at a dose of 200 mg/kg for 15 days followed by FST and TST. Group-4, considered as test drug treatment-2, in which *H. annuus* floret extract at a dose of 400 mg/kg was administered for 15 days followed by FST and TST. Group-5, considered as standard drug treatment, in which fluoxetine with a dose of 20 mg/kg was administered for 15 days followed by FST and TST [19].

### 2.7 Development of depression in animal models

#### 2.7.1 FST

**Figure 2:**
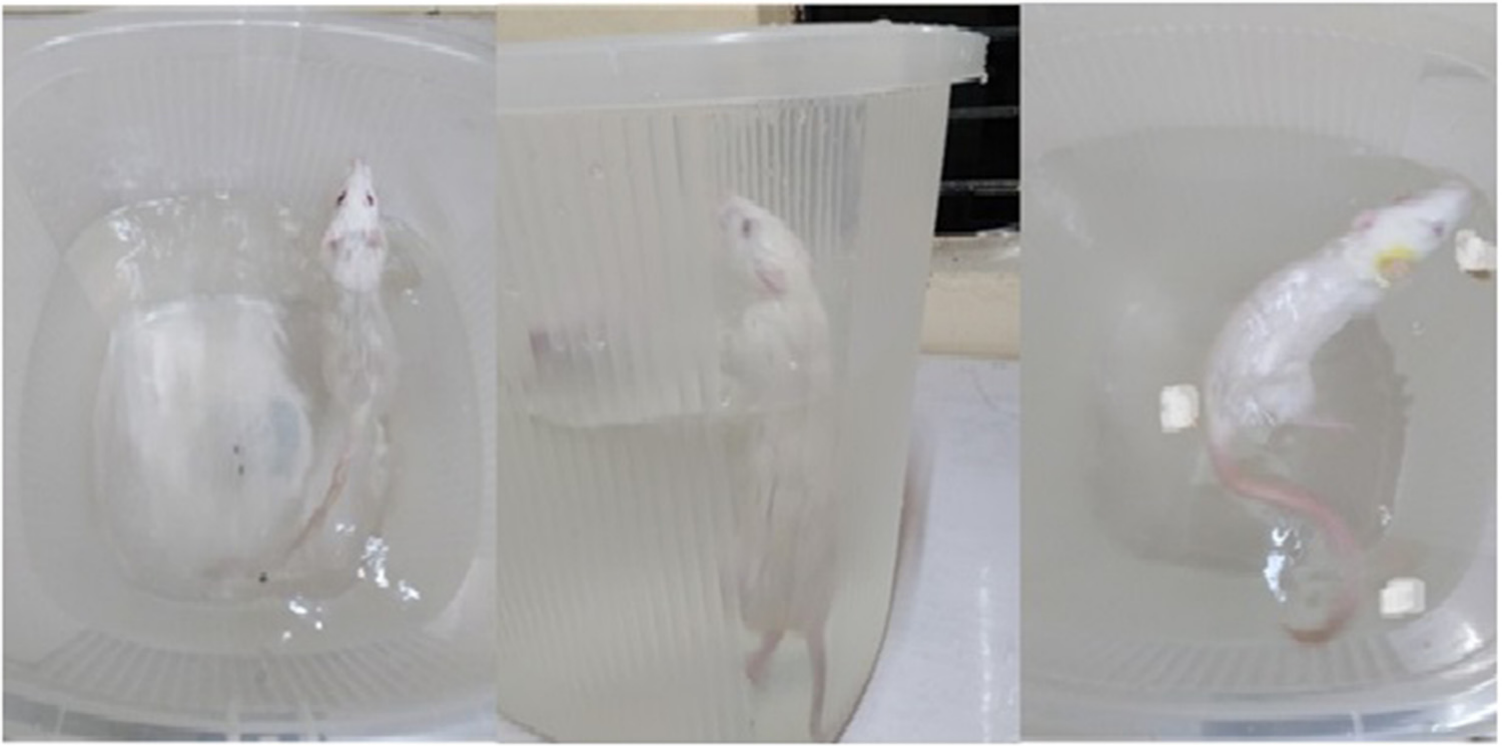
Illustrate FST (immobility, climbing, and swimming times).

The test protocol was executed in accordance with the procedure outlined by Porsolt et al. [20] to induce depression, which is illustrated as Figure 2. Briefly, rats were individually placed in transparent beakers (height: 60 cm; diameter: 20 cm) filled with water up to 40 cm height and maintained at a temperature of 25°C and forced to swim. The immobility time for each rat was measured for a 5 min test period. The entire stagnation duration is defined as the time duration in which the animals remained motionless or performed just the smallest limb movements required for floating. Immobility is defined as depression-like conduct. One hour before performing the experiment, the extract of *H. annuus* (200 and 400 mg/kg) and fluoxetine (20 mg/kg) were administered.

#### 2.7.2 TST

**Figure 3:**
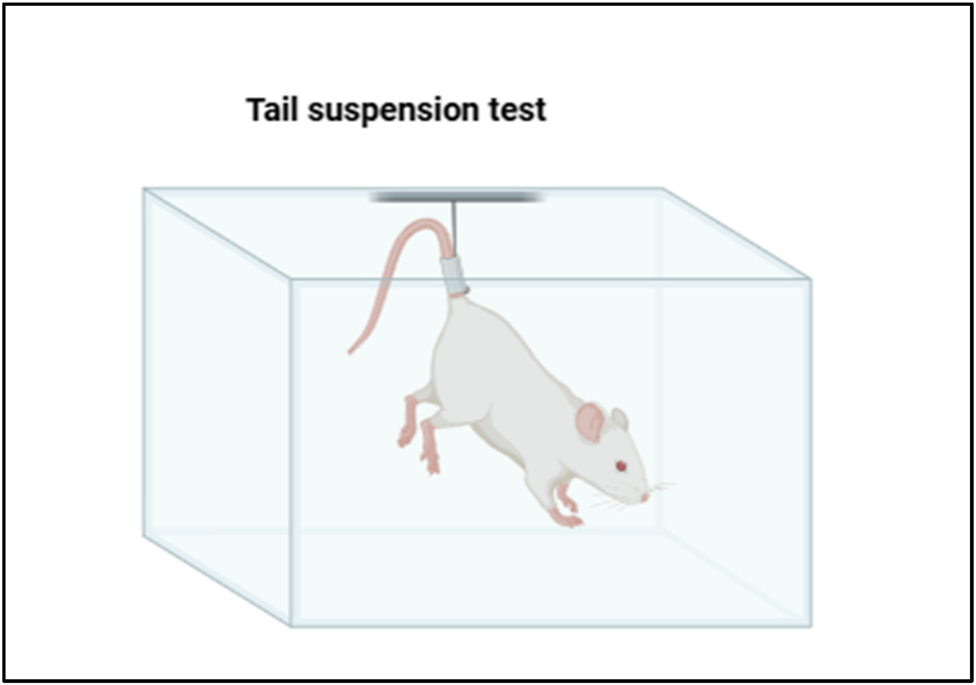
Representative diagram of TST.

The TST was done by the method described by Steru et al. [21] to produce depression in rats (Figure 3). The rats were positioned with their tail approximately 60 cm above the table’s surface using adhesive tape. The time of immobility was tracked for a period of 5 min. Rats were deemed immobile when they showed no sign of activity of their body parts and were hanging passively. One hour before performing the experiment, the extract of *H. annuus* (200 and 400 mg/kg) and fluoxetine (20 mg/kg) were administered.

### 2.8 Blood collection and organ isolation

At the end of the behavioural study, rats were anaesthetized after injecting thiopental sodium (40 mg/kg) intraperitoneally to collect blood samples. The collected blood was kept aside for 15 min and then centrifuged at 3,000 rpm for 10 min to isolate serum. The separated serum was used for cytokine estimation (TNF-α and IL-6). After blood collection, rats were sacrificed by exsanguination, and the brain of each rat was removed for biochemical estimations.

### 2.9 Homogenate preparation

Brain tissue homogenate was prepared using a phosphate buffer solution (pH 7.4) having protease inhibitors (1 µg/ml). Initially, the tissue was crushed using a homogenizer in phosphate buffer and protease inhibitor, and then it was centrifuged for 5 min at 1,000 g with the temperature maintained at 4°C. The supernatant formed was then transferred into another tube, which was used for lipid peroxidation (LPO) and GSH estimation. The remaining supernatant was recentrifuged for 15 min at 10,500 g at 4°C to get the post-mitochondrial supernatant (PMS). The PMS was then utilized further to measure the superoxide dismutase (SOD) and catalase (CAT) activity.

### 2.10 Oxidative stress parameters

To ascertain each sample’s protein concentration, Lowry et al.’s method was used [22]. Estimation of malondialdehyde (MDA) was done through the procedure described by Ohkawa et al. [23]. MDA is an end product of LPO and is a symbolic marker of oxidative stress. An assay with antioxidant enzymes like SOD was carried out by the methodology of Kono [24]. SOD is an essential antioxidant enzyme, and its function is to protect cell from superoxide radical stress. The estimated value of SOD is based on its capacity to stave off the reduction of NBT. CAT was estimated by the methodology described by Lück [25]. CAT promotes the conversion of H_2_O_2_ to water and oxygen. Reduced GSH was estimated through the methodology described by Jollow et al. [26].

### 2.11 Neurotransmitter estimation

#### 2.11.1 Estimation of NA and DA

For the estimation of neurotransmitters, first, homogenate of the brain tissue sample was prepared according to the method described by Schlumpf et al. [27]. After that, neurotransmitters NA and DA were estimated by the procedure described by Holm et al. [28]. About 0.2 ml of aqueous phase of homogenate was taken in 0.1 ml of EDTA/sodium acetate buffer (pH 6.9), and then 0.05 ml of 0.4 M HCl and 0.1 ml of iodine solution (0.1 M in ethanol) were added for oxidation. After 2 min, the reaction was stopped by adding 0.1 ml of Na_2_SO_3_ solution. After 1.5 min, 0.1 ml of acetic acid was added. The solution was then heated to 100°C for 6 min, and then the sample was allowed to reach at room temperature. Excitation and emission spectra were determined using a spectrofluorometer. The readings were taken at 395–485 nm for NA and 330–375 nm for DA.

#### 2.11.2 Estimation of 5-HT

Schlumpf et al.’s technique was used to determine the 5-HT concentration [27]. In this procedure, *o*-phthaldehyde reagent (0.25 ml) was mixed with tissue homogenate (0.2 ml), and the mixture was then heated at 100°C for 10 min in order to allow the fluorophore to be developed. The sample was allowed to reach room temperature, and then fluorescence readings were taken at 360–470 nm.

### 2.12 Assessment of inflammatory cytokines

Estimation of cytokines from serum was done by using sandwich ELISA kits as per instructions provided by the manufacturer.

### 2.13 Statistical analysis

A minimum of *n* = 6 animals per group was used, and the results of this research study were analysed by one-way ANOVA using GraphPad Prism version 10.2. The analysed results were presented as mean ± SEM. *P*-values less than 0.05 were deemed statistically significant.


**Ethical approval:** The research related to animals’ use complied with all the relevant national regulations and institutional policies for the care and use of animals. The research study protocol was scientifically reviewed and approved by the IAEC of HIMT College of Pharmacy in accordance with CCSEA (approval no. 2024/HIMT/IAEC/FB/007).

## 3 Results

### 3.1 Pre-emptive phytochemical assessment

The results of phytoconstituent assessment of *H. annuus* floret extract are described in Table 1.

**Table 1:** Phytoconstituent assessment of the *H. annuus* floret extract

Phytoconstituent	Test	Colour formation	Ethanol extract
Alkaloids	Mayers test	Creamy white precipitate	+
Flavonoids	Alkaline reagent test	Yellow tint turns colourless	+
Tannins	Ferric chloride test	Brownish colour and blue-black	±
Saponins	Froth test	Froth (foam)	±
Glycosides	Keller-Kailani test	Violet or bluish-green	±
Phenolics	Ferric chloride test	Green or bluish tint	±
Terpenoids	Salkowski test	Reddish-brown	+++
Steroids	Liebermann–Burchard test	Blue or green tint	+++

Keywords: (+) indicates presence, (±) indicates trace amounts, (++) indicates high presence, (+++) indicates very high presence, (−) indicates absence, and (±) trace amount. The ethanol extracts of *H. annuus* flowers (ray and disk florets) were shown to have significant quantities of alkaloids, flavonoids, saponin, glycosides, tannins, phenolics and terpenoids, and steroids, as indicated by the data in the above table.

### 3.2 Effect of *H. annuus* flower extract on the FST

The immobility, swimming, and climbing times were investigated in all groups of rats, as demonstrated in Table 2. Rats of group-2 demonstrated significant enhancement in immobility time and reduction in swimming and climbing time, as compared to group-1 rats (*p* < 0.0001). *H. annuus* extract administered at doses 200 and 400 mg/kg showed significant antidepressant activity by decreasing the immobility time and increasing the swimming and climbing times in rats of groups-3 and 4 compared to group-2 rats (*p* < 0.01, *p* < 0.001, and *p* < 0.0001), respectively. Group-5 rats in which standard drug was administered showed a significant decrease in immobility time and an increase in swimming and climbing times compared with group-2 rats (*p* < 0.0001).

**Table 2:** Effect of hydroalcoholic extract of *H. annuus* florets in the FST

Group	Treatment	Immobility time (s)	Swimming time (s)	Climbing time (s)
		Mean ± SEM	Mean ± SEM	Mean ± SEM
Group-1	Normal saline	37.318 ± 1.76	360.33 ± 2.56	92.54 ± 2.72
Group-2	Depression control	146.67 ± 4.41^####^	187.00 ± 2.64^####^	42.0 ± 2.88^####^
Group-3	*H. annuus* extract 200 mg/kg	78.50 ± 3.64****	215.33 ± 5.43***	61.0 ± 3.69**
Group-4	*H. annuus* extract 400 mg/kg	54.50 ± 2.79****	273.83 ± 2.28****	62.66 ± 2.66***
Group-5	Fluoxetine 20 mg/kg	40.50 ± 2.27****	266.66 ± 5.72****	71.0 ± 2.81****

Group-2 vs Group I ^####^ *p* < 0.0001; Group-3 vs Group 2. **p < 0.01; Group 4 vs Group 2 ***p < 0.001; Group-5 vs Group 2 → ****p < 0.0001.

### 3.3 Effect of *H. annuus* flower extract on the TST

The result of TST demonstrated the antidepressant activity of *H. annuus* extract (Table 3). Considerable escalation in immobility time (*p* < 0.0001) was seen in group-2 rats when it is compared with group-1. Treatment with hydroalcoholic extract of *H. annuus* showed notable diminution in immobility time (*p* < 0.01 and *p* < 0.0001) in groups 3 and 4 rats when compared with group 2 rats. Group-5 rats in which standard drug was administered showed notable (*p* < 0.0001) diminution in immobility time when it is compared with group-2 rats.

**Table 3:** Effect of hydroalcoholic extract of *H. annuus* florets in the TST

Group	Treatment	Immobility time (s) Mean ± SEM
Group-1	Normal saline	30.43 ± 1.98
Group-2	Depression control	161.77 ± 4.26^####^
Group-3	*H. annuus* extract 200 mg/kg	138.84 ± 5.85**
Group-4	*H. annuus* extract 400 mg/kg	82.01 ± 4.40****
Group-5	Fluoxetine 20 mg/kg	48.82 ± 3.93****

Group-2 vs Group I → ^####^ *p* < 0.0001; Group-3 vs Group 2 → **p < 0.01; Group 4 vs Group 2 → ****p < 0.0001; Group-5 vs Group 2 → ****p < 0.0001.

### 3.4 Effect of *H. annuus* flower extract on neurotransmitter estimation

#### 3.4.1 5-HT

The level of 5-HT in the brain tissue was found to be significantly reduced (*p* < 0.0001) in group-2 when compared with group-1. Administration of *H. annuus* floret extract in groups-3 and 4 at doses of 200 and 400 mg/kg notably elevated (*p* < 0.0001) the amount of 5-HT in the brain tissue when compared with group-2. Administration of standard drug at a dose of 20 mg/kg in group-5 markedly elevated (*p* < 0.0001) the 5-HT level when compared with group-2 (Table 4).

#### 3.4.2 DA

DA concentration was markedly reduced (*p* < 0.0001) in the brain tissue of group-2 when compared with group-1. Administration of *H. annuus* floret extract in groups-3 and 4 at doses of 200 and 400 mg/kg notably improved (*p* < 0.001 and *p* < 0.0001) the level of DA when compared with group-2. Standard drug administration at a dose of 20 mg/kg in group-5 considerably improved (*p* < 0.0001) the DA level when compared with group-2.

#### 3.4.3 NE

The level of NE in the brain tissue was found to be significantly reduced (*p* < 0.0001) in group-2 when compared with group-1. Administration of *H. annuus* floret extract in group 3 showed non significant result, whereas in group 4 showed markedly elevated (*p* < 0.001) amount of NE in the brain tissue when compared with group 2. Administration of standard drug at a dose of 20 mg/kg in group-5 notably elevated (*p* < 0.0001) the NE level when compared with group-2.

### 3.5 Oxidative stress parameters

#### 3.5.1 LPO

**Figure 4:**
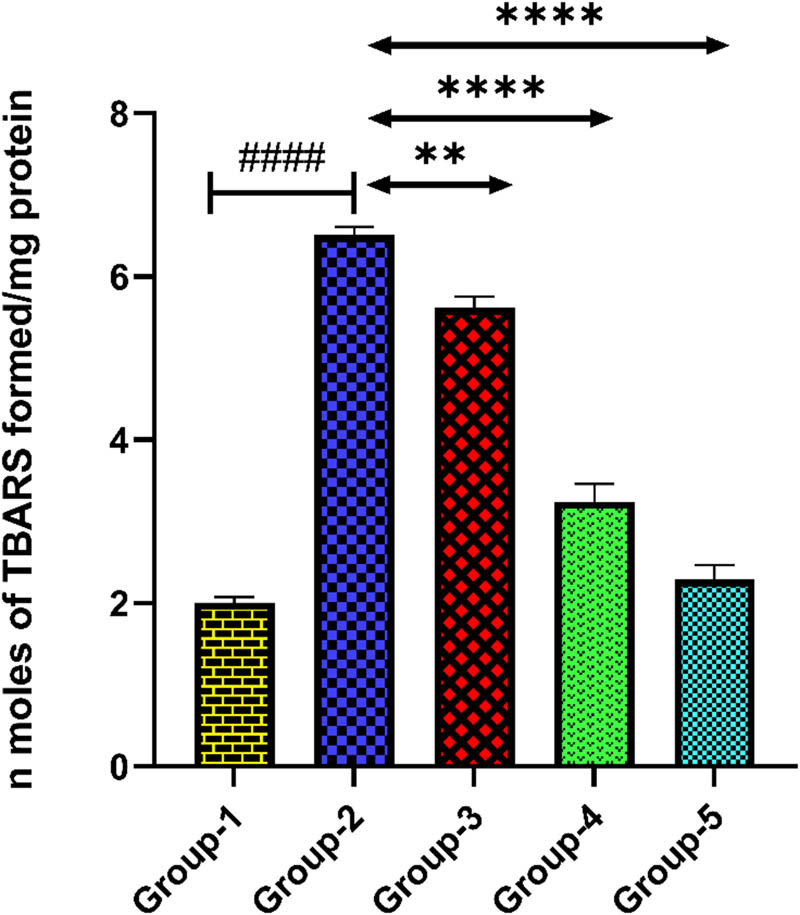
Level of MDA in the rat brain tissue. Group-2 shows → ^####^ *p* < 0.0001 when it is compared with group 1. Group-3 shows → **p < 0.01 and group 4 shows → ****p < 0.0001 when it is compared with the diseased group. Group-5 shows → ****p < 0.0001 when it is compared with group 2.

Depression control group (group-2) showed higher level of MDA in the brain tissue when it is compared with group-1 (*p* < 0.0001). *H. annuus* floret extract administration at doses 200 and 400 mg/kg in treatment control groups (3 and 4) significantly depleted the MDA level when compared with the depression control group (*p* < 0.01, *p* < 0.0001). Administration of standard drug at dose of 20 mg/kg in group-5 significantly reduced the MDA level in the brain tissue when compared with group-2 (*p* < 0.0001) (Table 4 and Figure 4).

**Table 4:** Effect of hydroalcoholic extract of *H. annuus* florets on the neurotransmitter level

Group	Treatment	5-HT (µg/g tissue)	DA (µg/g tissue)	NE (µg/g tissue)
Group-1	Normal saline	103.19 ± 2.43	107.63 ± 3.96	85.20 ± 3.75
Group-2	Depression control	46.58 ± 3.74****	49.50 ± 2.94****	48.83 ± 1.49****
Group-3	*H. annuus* extract 200 mg/kg	63.44 ± 4.88***	74.17 ± 3.67***	59.69 ± 1.97 ns
Group-4	*H. annuus* extract 400 mg/kg	85.69 ± 5.47****	88.28 ± 2.75****	69.24 ± 3.09***
Group-5	Fluoxetine 20 mg/kg	91.16 ± 3.79****	100.54 ± 4.16****	78.86 ± 3.33****

Group-2 vs Group I → ^####^ *p* < 0.0001; Group-3 vs Group 2 → ***p < 0.001. ns *p* < 0.05; Group 4 vs Group 2 → ****p < 0.0001; Group-5 vs Group 2 → ****p < 0.0001.

#### 3.5.2 Reduced GSH

**Figure 5:**
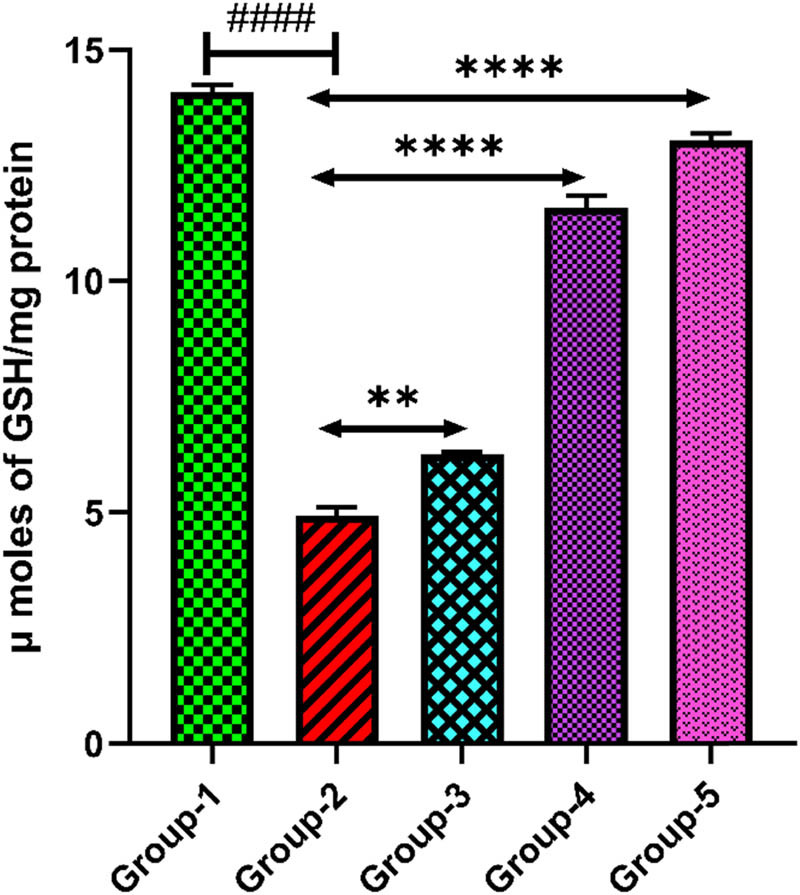
Level of GSH in the rat brain tissue. Group-2 shows → ^####^ *p* < 0.0001 when it is compared with group-1. Group-3 shows → **p < 0.01 and group 4 shows → ****p < 0.0001 when compared with group-2. Group-5 when compared with group-2 shows → ****p < 0.0001.

GSH level in the brain tissue was found to be reduced in group-2 when compared with group-1 (*p* < 0.0001). *H. annuus* floret extract administration at doses 200 and 400 mg/kg in treatment control groups-3 and 4 significantly (*p* < 0.01, *p* < 0.0001) elevated the GSH level compared to group-2. Administration of standard drug at dose of 20 mg/kg in group-5 notably elevate the GSH level in brain tissue when compared with group-2 (*p* < 0.0001) (Figure 5).

#### 3.5.3 SOD

**Figure 6:**
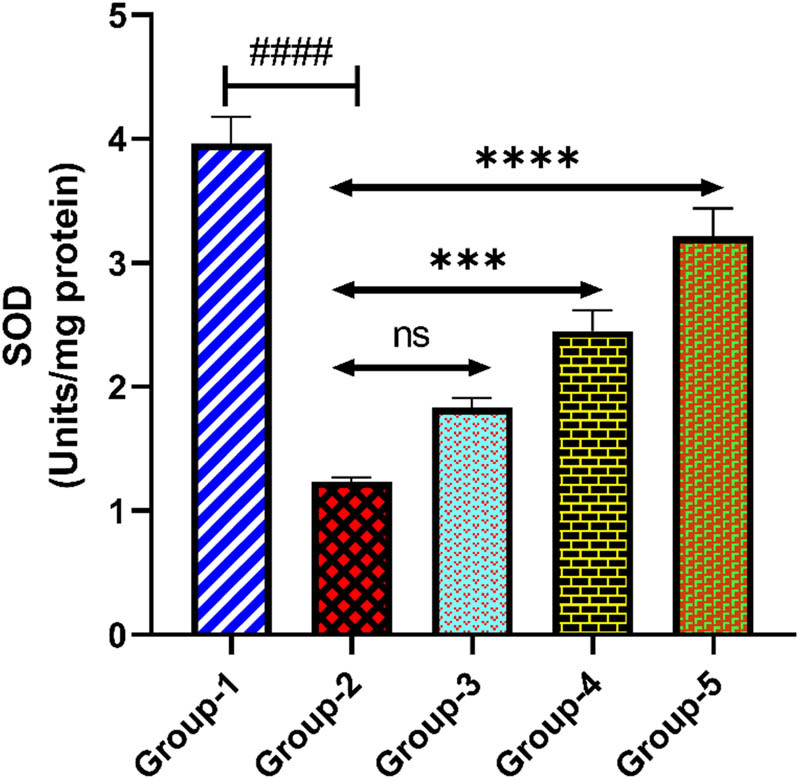
Level of SOD in the rat brain tissue. Group-2 shows → ^####^ *p* < 0.0001 when it is compared with group-1. Group-3 shows → ^ns^ *p* > 0.05, and group 4 shows → ***p < 0.001 when compared with group-2. Group-5 when compared with group-2 shows → ****p < 0.0001.

The level of SOD in the brain tissue was found to be reduced in group-2 when it was compared with group-1 (*p* < 0.0001). Administration of *H. annuus* floret extract in group-3 (200 mg/kg) did not considerably elevate the level of SOD when compared with group-2 (*p* > 0.05). However, *H. annuus* floret extract in group-4 (400 mg/kg) notably elevated the level of SOD when it was compared with group-2 (*p* < 0.001). Standard drug administration at a dose of 20 mg/kg in group-5 notably elevated the SOD level compared to the diseased group (*p* < 0.0001) (Figure 6).

#### 3.5.4 CAT

**Figure 7:**
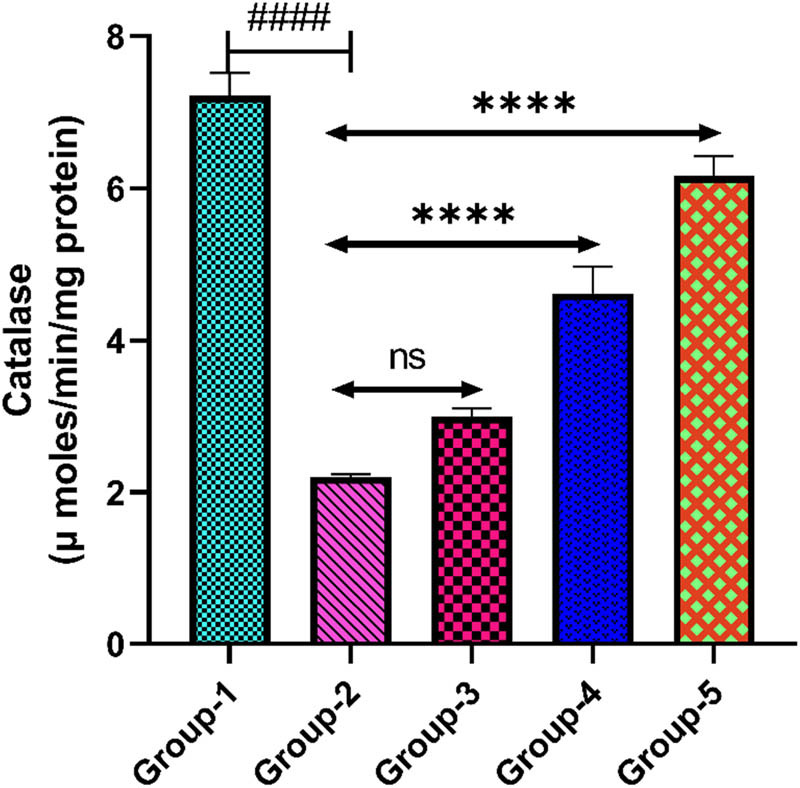
Level of CAT in the rat brain tissue. Group-2 shows → ^####^ *p* < 0.0001 when it is compared with group-1. Group-3 shows → ^ns^ *p* > 0.05, and group 4 shows → ****p < 0.0001 when compared with group-2. Group-5 when compared with group-2 shows → ****p < 0.0001.

The depression control group showed a lower level of CAT in the brain tissue when it was compared with group-1 (*p* < 0.0001). Administration of *H. annuus* floret extract in group-3 (200 mg/kg) did not notably elevate the level of CAT when it was compared with group-2 (*p* > 0.05). However, *H. annuus* floret extract in group-4 (400 mg/kg) notably elevated the level of CAT when it was compared with group-2 (*p* < 0.0001). Standard drug administration at a dose of 20 mg/kg in group-5 notably elevated the CAT level compared to group-2 (*p* < 0.0001) (Figure 7).

### 3.6 Cytokine estimation

#### 3.6.1 IL-6

**Figure 8:**
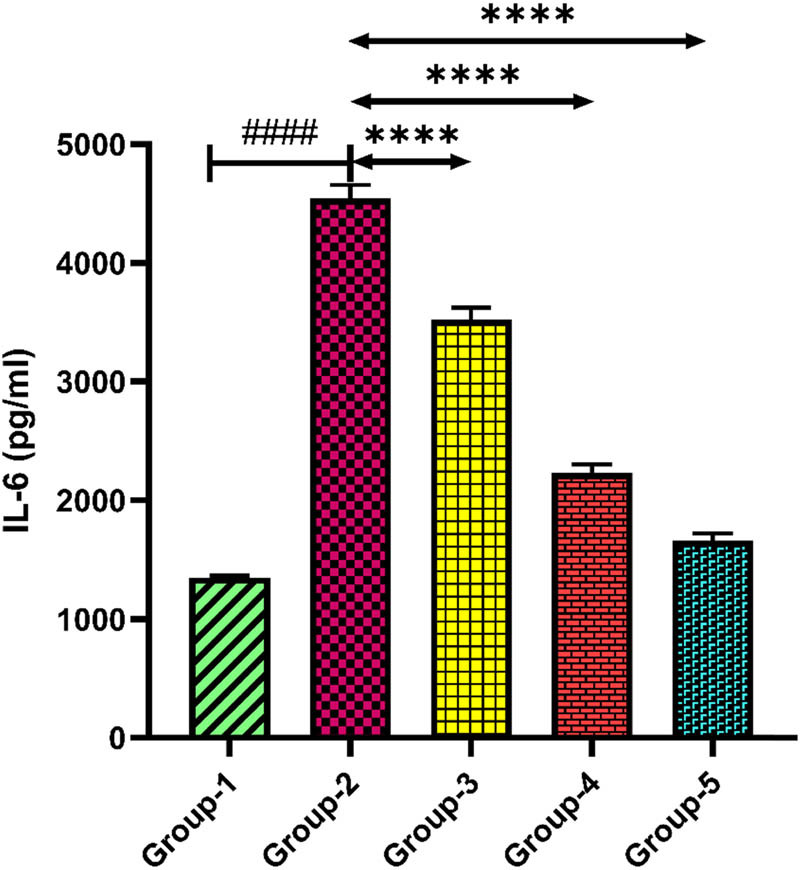
Level of IL-6 in the serum sample. Group-2 shows → ^####^ *p* < 0.0001 when compared with normal control. Groups-3 and 4 show → ****p < 0.0001 when compared with group 2. Group-5 when compared with group 2 shows → ****p < 0.0001.

The level of inflammatory cytokine IL-6 was found to be elevated in group-2 when compared with group-1 (*p* < 0.0001). *H. annuus* floret extract administration in groups-3 and 4 at doses of 200 and 400 mg/kg notably reduced the IL-6 level when compared with group-2 (*p* < 0.001). Standard drug fluoxetine at a dose of 20 mg/kg in group-5 reduced the level of IL-6 when compared with group-2 (Figure 8).

#### 3.6.2 TNF-α

**Figure 9:**
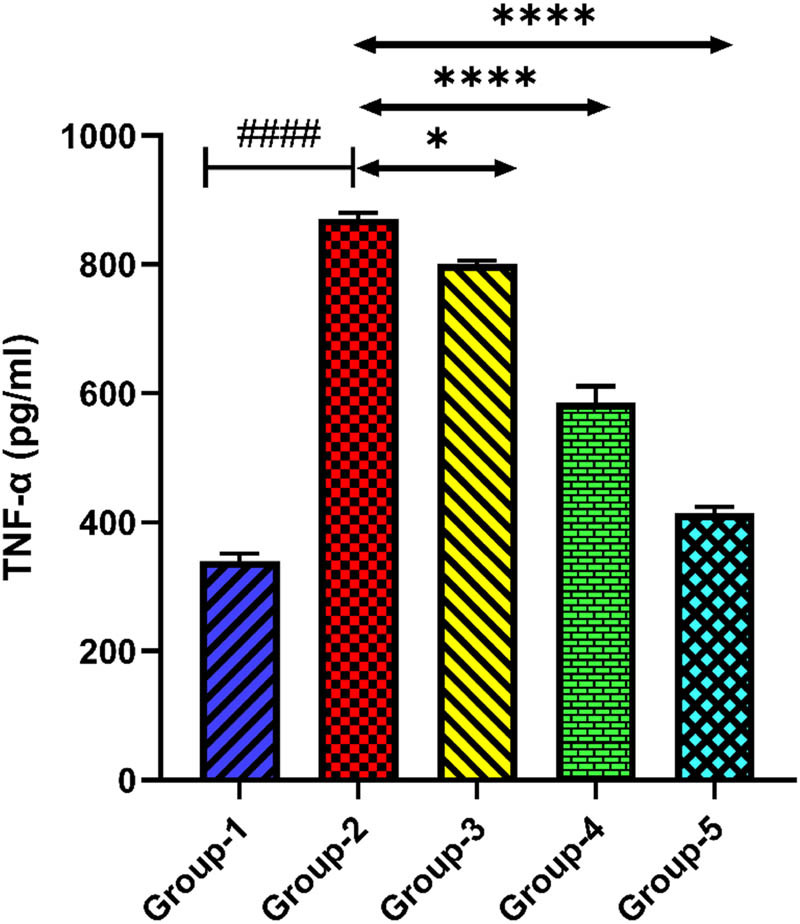
Level of TNF-α. Group-2 shows → ^####^ *p* < 0.0001 when compared with normal control. Group-3 shows → *p < 0.05 and group 4 shows → ****p < 0.0001 when compared with group 2. Group 5 when compared with group 2 shows → ****p < 0.0001.

The level of TNF-α was also detected to be elevated in group-2 when compared with group-1 (*p* < 0.0001). Administration of *H. annuus* floret extract at doses of 200 and 400 mg/kg to groups-3 and 4 significantly (*p* < 0.05, *p* < 0.0001) reduced the level of TNF-α when compared with group-2. Standard drug fluoxetine at a dose of 20 mg/kg in group-5 reduced the level of TNF-α when compared with group-2 (Figure 9).

## 4 Discussion

MDD, for instance, affects over 17% of people in their lifetime and poses a serious threat to the public health. It is anticipated that, by 2030, it will be the second leading cause of disability globally, surpassing cardiovascular diseases [29]. A previous research study revealed that triterpene glycosides found in sunflower (*Helianthus annuus*) exhibit anti-inflammatory activity [30]. Ethanolic extract of leaves of *Helianthus annuus* demonstrated anti-spermatogenic activity by alleviating degenerative changes in the testes, epididymal sperm properties, and testosterone levels [31]. The essential oils present in sunflower exhibited antifungal, antibacterial, and antioxidant activities, and the major constituents are α-pinene (26.00%), verbenone (7.40%), terpinolene (1.69%), and α-terpineol (1.27%) [32].

The pathophysiology of depression now emphasizes the role of inflammation and the immune system. Increased levels of inflammatory mediators, such as cytokines like IL-1β, TNF-α, and IL-6, are often seen in patients who suffer from severe depression [33,34]. These markers are not only contributors to the illness process but also serve as markers of inflammation. Previous researchers have evaluated the role of inflammatory mediators (cytokines and chemokines) and C-reactive proteins in patients of depression. Studies on animals have demonstrated that these cytokines can cause depression by inducing depressive behaviours on repeated exposure, demonstrating the reciprocal link between inflammation and depression [35]. Inflammatory cytokines have been linked to depression by interfering with brain plasticity, neuroendocrine function, and neurotransmitter metabolism. For instance, it has been demonstrated that systemic circulation of IL-1, IL-2, and IL-6 considerably alters brain 5-HT and DA levels. It has been found that impaired central dopaminergic neurotransmission is linked with diminished release of DA and or reduced expression of DA receptors, which possibly regulates the release of inflammatory cytokines and associated with depression. Proinflammatory cytokines also exacerbate depressive symptoms by altering NE activity in the locus coeruleus, hippocampus, and hypothalamus, among other brain regions [36,37,38].

It is interesting to note that several antidepressants have anti-inflammatory properties, which could explain part of their effectiveness. Examples of substances that have both anti-inflammatory and antidepressant properties include herbs like *Withania somnifera* and *Curcuma longa*, as well as herbal components like hyperforin from St. John’s wort [39,40]. Inflammatory system participation in the symptomatology of depression, which spans the emotional and biological domains, requires a comprehensive approach to both diagnosis and treatment. Pessimistic attitudes, strong emotions like apathy, unhappiness, and remorse, and a skewed self-perception that is frequently typified by feelings of ugliness are examples of emotional symptoms. These symptoms usually accompany a lack of drive, which makes it challenging to make even seemingly straightforward judgments [41,42].

*H. annuus* provides a novel opportunity for exploring natural antidepressant treatment that may integrate traditional neurotransmitter concepts with new insights into inflammation. It may be therapeutically effective, given its capacity to alter depressive biomarkers and behavioural reactions. To confirm these results, we investigated the complete therapeutic potential of *H. annuus* in the treatment of depressive illnesses as well as to establish the precise mechanisms of action. Numerous chemicals, including alkaloids, flavonoids, tannins, saponins, glycosides, phenolics, terpenoids, and steroids, were found in the phytochemical screening process. Steroids and terpenoids are highly prevalent, suggesting that these substances may be essential to the biological processes that have been observed [43]. The hydroalcoholic extract of *H. annuus* has antidepressant-like qualities, as demonstrated by decreased immobility periods and increased swimming and climbing times in the FST. The greater dose demonstrated more robust antidepressant effects, similar to fluoxetine, in a dose-dependent fashion [44]. Rats administered with the hydroalcoholic extract of *H. annuus* had shorter immobility periods in the TST, which suggests a reduction in depressive-like behaviour. The extract had a dose-dependent impact, with larger doses showing greater effects. The extract’s antidepressant abilities were validated by the notable efficacy of fluoxetine.

In recent research, the pathogenesis of depression demonstrated significant decline in monoamine neurotransmitters (5-HT, NA, and DA) or dysfunction in secondary messengers. Traditional treatments have mostly concentrated on modifying functions of these systems. Low 5-HT levels are generally related to emotional symptoms like anhedonia, sorrow, indecision, and suicidal thoughts, whereas low NE levels are linked to bodily symptoms like irregular sleep patterns, changes in appetite, and fluctuations in libido [45]. Low levels of these monoaminergic neurotransmitters play a significant role in the pathophysiology of depression. 5-HT is a key neurotransmitter in mood control as well as in neuroplasticity in the initial phases of neural development [46]. The validity of the models in simulating depressive conditions is demonstrated by the significant reduction in 5-HT levels observed in the depression control group. The hydroalcoholic extract of HA demonstrated a considerable restorative effect on 5-HT levels. This implies that the HA extract might have stimulated serotonergic neurons to increase the level of 5-HT, which could be involved in its possible antidepressant benefits.

DA is imperative in addressing the pleasure deficits of depressed persons. Previous studies have highlighted the impact of dopaminergic system disruptions in depression, which often involve impaired DA release or altered function or reduced expression of dopaminergic receptors [47]. Dopaminergic dysregulation linked to depression is highlighted through the notable reduction in the DA level observed in the depression control group. DA levels were shown to rise in response to the hydroalcoholic extract of HA at both dosages; however, the larger dose had a more pronounced effect. DA levels were also markedly elevated by fluoxetine, emphasizing the drug’s complex mode of action. The capacity of the HA extract to raise DA levels indicates that it may be useful in treating depressive symptoms associated with dopaminergic deficiencies.

NE is also an essential neurotransmitter for controlling mood, arousal, and attention [48]. The catecholaminergic imbalance associated with depression is highlighted by the significant drop in NE levels observed in the depression control group. NE levels were markedly elevated by the HA extract, particularly at a higher dose, suggesting that the extract may be able to restore the catecholaminergic function. The fact that fluoxetine can raise NE levels considerably adds to the evidence, supporting its efficacy as a wide-acting antidepressant.

Oxidative stress commences due to inequality between the production of ROS/RNS and the capacity of body organs to regulate them through enzymatic and non-enzymatic defence mechanisms. Our brain is very sensitive to oxidative damage due to the generation of high levels of ROS/RNS. A significant rise in oxidative stress parameters decreases the effectiveness of antioxidative defence mechanisms, which further compromises neuronal safeguarding. As a result, variations in the expression and functionality of pro- and antioxidative enzymes have been documented by several researchers [49].

MDA is a final product formed in LPO that is caused due to oxidative stress and found in higher concentrations of depressed patients [50]. Remarkably, MDA levels were found to be higher in the depression control group. This could indicate an increase in LPO. When compared with the treatment and standard control groups, MDA levels were somewhat reduced by fluoxetine and HA extract dosages, but they were still higher than those of the normal group. This suggests that the HA extract has antioxidant properties that reduce the LPO. GSH is a major antioxidant that protects cells from oxidative damage, and in this process GSH is converted into oxidized glutathione (GSSG), which is further restored by GSH peroxidase [51]. Consequently, the reduced level of GSH in the depression control group is an indicative of an oxidative stress response. GSH levels were markedly improved by fluoxetine and HA extract doses, indicating a reduction in oxidative stress. It is clear that the HA extract has a dose-dependent impact, with higher dosages demonstrating better efficacy. Fluoxetine’s strong antioxidant qualities are highlighted by the significant increase in GSH levels.

On the other hand, SOD is an essential antioxidative enzyme which decomposes the superoxide anion to O_2_ and H_2_O_2_, and it has been found to be reduced in depressed individuals [52]. A compensating reaction to increased oxidative stress is shown in the depression control group, showing lower SOD activity. Effective oxidative stress mitigation is suggested by the improvement of SOD activity towards normal levels observed in both fluoxetine and HA extract dosages. Stronger antioxidant effects were seen at a higher dosage of HA (400 mg/kg), which was consistent with the improvement in oxidative indicators. CAT represents a key antioxidant enzyme that declines the effects of oxidative stress via fragmenting H_2_O_2_ into H_2_O and O_2_ [53,54]. The depression control group decreased CAT activity, which demonstrates an adaptive reaction to oxidative stress. CAT activity was successfully raised to normal levels by fluoxetine and HA extract doses, suggesting a decrease in oxidative stress. The antioxidant effect of HA extract was further supported by CAT activity, and a similarity between fluoxetine and the higher dosage of HA extract has been observed.

In response to oxidative damage, the inflammatory processes accelerate the production of proinflammatory cytokines, demonstrating depressive behaviours in patients [55]. Thus, the roles of both oxidative damage and neuroinflammation are important in the onset of depression. A previous research study reported that the leaf extract of *Helianthus annuus* attenuated atopic dermatitis (AD) by modulating inflammatory cytokines [56]. The elevated IL-6 levels verify the existence of neuroinflammation linked to depressive states in the depression control group. The strong anti-inflammatory qualities of HA extract are demonstrated by the considerable reduction in IL-6 levels observed after treatment with both dosages. The HA extract demonstrated a dose-dependent impact, which is clearly evident in our investigation, and it was identified that greater dosages are more effective in reducing neuroinflammation. The well-known antidepressant, such as fluoxetine also showed a significant reduction in IL-6 levels, which is consistent with its antioxidant and anti-inflammatory properties. TNF-α is an additional important pro-inflammatory cytokine that contributes significantly to the inflammatory response and is linked to the aetiology of depression [57,58]. The occurrence of neuroinflammation in the depressive state is further confirmed by the higher TNF-α levels in the depression control group. HA extract treatment at both dosages resulted in a considerable decrease in TNF-α levels, showing its strong anti-inflammatory effects. The dose-dependent effect, in which a higher dosage demonstrates greater efficacy, supports the possibility of HA extract in attenuating neuroinflammation. The significant decrease in TNF-α levels observed with HA and fluoxetine accentuates its potent anti-inflammatory properties, which support its effectiveness as a treatment alternative for depression.

## 5 Conclusions

This study aims to investigate the antidepressant effects of hydroalcoholic extracts from HA florets in animal models. Based on both behavioural and biochemical evaluations, the results showed that the sunflower extract has considerable antidepressant effects. Rats treated with HA extract showed shorter immobility periods during the FST and TST, which suggested a decrease in depression-like behaviour. Swimming and climbing were among the more energetic activities that were noted, which may indicate increased motivation and less hopelessness. Its potential natural antidepressant property was further supported by comparison with fluoxetine, which revealed that the HA extract had similar effects. 5-HT, DA, and NE levels were higher in the groups treated with HA extract, according to neurotransmitter analysis. Since these neurotransmitters play a crucial role in mood regulation, traditional antidepressants frequently target them. Rats receiving treatment had improved profiles for oxidative stress markers, including lower levels of MDA and higher activity of antioxidant enzymes like CAT, GSH, and SOD. This shows that by lowering oxidative stress, which is frequently linked to depression, the extract may demonstrate potential neuroprotective effects by counteracting depression.
